# Investigation of predictors of body awareness of pregnant women

**DOI:** 10.1590/1806-9282.20241931

**Published:** 2025-07-07

**Authors:** Aslıhan Turan, Hasan Gerçek, Hafize Dağ Tüzmen, Jule Eriç Horasanli

**Affiliations:** 1 Konya Chamber of Commerce Karatay University, Faculty of Health Sciences, Department of Midwifery – Konya, Turkey.; 2 Konya Chamber of Commerce Karatay University, Vocational School of Health Sciences, Department of Therapy and Rehabilitation – Konya, Turkey.; 3 Necmettin Erbakan University, Faculty of Medicine, Department of Gynaecology and Obstetrics – Konya, Turkey.

**Keywords:** Body image, Gestational age, Physical activity, Pregnancy

## Abstract

**OBJECTIVE:**

The aim of this study was to determine the predictors of body awareness in pregnant women.

**METHODS:**

A cross-sectional study was conducted with 149 women in their second and third trimesters of pregnancy in Turkey between August and November 2024. Participants completed a sociodemographic form, the Body Awareness Questionnaire, and the International Physical Activity Questionnaire-Short Form. Multiple linear regression was used to analyze the effects of age, number of pregnancies, gestational weeks, and physical activity levels on body awareness.

**RESULTS:**

Participants had a mean age of 27.69±4.82 years, a mean body mass index of 26.59±3.49, and a mean gestational week of 31.97±6.95. The average body awareness score was 92.52±15.48, and the mean physical activity level was 1,084.56±671.93 metabolic equivalent of task-minutes/week. Regression analysis showed that gestational weeks significantly predicted body awareness (β=-0.327, p<0.001), with decreased body awareness observed as the pregnancy progressed. No significant associations were found with age, number of pregnancies, or physical activity levels (p>0.05).

**CONCLUSION:**

Gestational weeks negatively influence body awareness in pregnant women. These findings highlight the importance of developing interventions to enhance body awareness during advanced stages of pregnancy.

## INTRODUCTION

Body awareness refers to an individual's more specific awareness of specific body parts by focusing attentively on their internal sensations of their own body^1^. Another concept related to body awareness is "body image". This concept implies that body awareness involves an external and visual perception channel. Body image has been studied in a wide range of fields, including psychiatry (e.g., anorexia), feminist psychology (e.g., self-objectification), and neuroscience (e.g., rubber hand illusion and amputation), as well as in upper extremity lymphedema after breast cancer surgery, reflecting the priority given to visual appearance rather than internal perceptions of the body^2,3^. Body awareness is affected by many variables such as age, number of pregnancies, and gestational age^4,5^.

Pregnancy causes significant physical changes in women's bodies. These rapid physical changes can cause women to reassess their body image^6^. Pregnancy can affect women's psychological health, including body image and quality of life^7^. Higher levels of body dissatisfaction during pregnancy, leading in particular to a lower level of trust in bodily signals (trust in the body) and a reduced ability to attend to and listen to bodily sensations^8^. As the pregnancy progresses, the displacement of the center of gravity and lumbar lordosis increases^9^, which can have a negative impact on body awareness.

Physical activity and exercise are gaining importance as one of the tools to improve body awareness, and it is also an effective method to increase self-efficacy and self-esteem^10,11^. It is known that physical activity during pregnancy is generally safe for maternal and fetal health and that physical activity during this period reduces the risk of adverse health outcomes for mother and fetus^12^. Exercise has been reported to reduce risk factors for body image dissatisfaction such as stress, social support, or body mass index^6^.

Although there are studies in the literature on the factors affecting body awareness of pregnant women, we did not find any studies examining the effects of age, number of pregnancies, gestational week, and physical activity level^1,7,8,13^. The hypothesis of this study was that increasing age, number of pregnancies, and gestational week decreased body awareness in pregnant women, while physical activity level increased body awareness. The aim of this study was to determine the relationship between the body awareness of pregnant women and physical activity levels, age, number of pregnancies, and gestational week.

## METHODS

**Figure 1:**
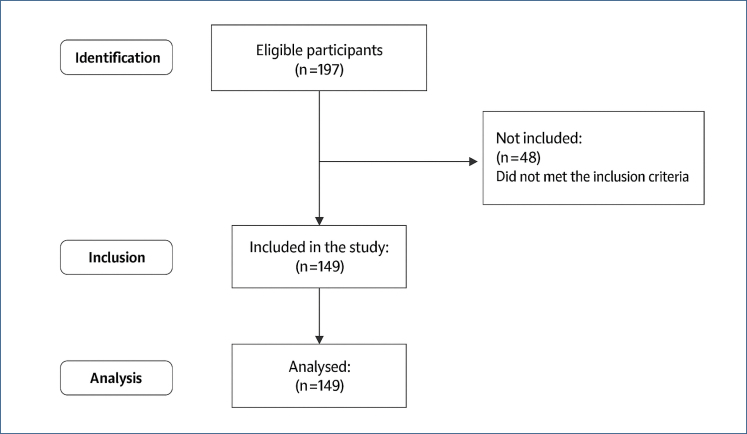
Flow diagram.

This study is a cross-sectional study conducted online via Google Forms between September and November 2024 to determine the effect of age, number of births, gestational week, and physical activity levels on body awareness of pregnant women in Turkey in accordance with the Declaration of Helsinki. A total of 197 people were screened, and 149 women in the second to third trimester of pregnancy (be at least 14 weeks of gestation) were included in the study (Figure 1). Prior to the study, ethical approval was obtained from the local ethics committee of KTO Karatay University [decision number: 2024/038 and prospectively registered at www.clinicaltrials.gov (NCT06648993)]. All participants provided informed consent prior to enrolling in this study. This study has been reported according to the Strengthening the Reporting of Observational Studies in Epidemiology (STROBE) statement to assist in quality reporting.

The inclusion criteria of the study were being 18–40 years old, being in the second to third trimester of pregnancy, and volunteering to participate in the study. The exclusion criteria for the study were having a high-risk pregnancy (preeclampsia, threatened preterm labor, gestational diabetes mellitus, etc.), multiple pregnancy, being prohibited from physical activity by the physician, being diagnosed psychiatric disorders (e.g., eating disorders and major depression), neurological conditions affecting sensory or motor function, chronic musculoskeletal pain, and extensive prior experience in body-awareness-based practices (e.g., yoga and Pilates).

### Sample size

G*Power software package (G*Power, Version 3.0.10, Franz Faul, Universität Kiel, Germany) was used to calculate the sample size. A minimum of 89 participants should be included in the study with an effect size of 0.15, which is the medium effect size for the linear multiple regression model, 95% power, and 0.05 Type I error. A total of 149 pregnant women were included to increase the power of the study. The post hoc power of the study was 0.95 with a Type I error of 0.05 and an effect size of 0.09.

### Outcome measures

Participants’ age, body mass index, number of pregnancies, number of live births, gestational weeks, educational status, income status, and employment status were collected using a sociodemographic form created by the researchers.

#### Body awareness

Body Awareness Questionnaire was used to evaluate body awareness. The Body Awareness Questionnaire is valid and reliable and has no cutoff point. The questionnaire consists of 18 questions. Participants were asked to score each statement ranging from 1 (1: Not true at all) to 7 (7: Completely true), and the total score was obtained by summing the score given for each statement. The total score takes a value between 18 and 126. The higher the total score, the better the body awareness^14,15^.

#### Physical activity

The physical activity levels of individuals were assessed using the IPAQ-SF, a tool comprising seven questions that gather data on the frequency (in days) and duration (in minutes) of high-, moderate-, and low-intensity activities over the previous week, as well as sedentary behavior. To be included in the scoring, an activity must be sustained for at least 10 min per session. The evaluation involves multiplying the reported minutes and days by the corresponding metabolic equivalent of task (MET) value, with the resulting score expressed as MET-minutes/week. Lower scores indicate lower levels of physical activity^16,17^.

#### Statistic

The normality of the distribution of values was examined using visual and analytical methods. As descriptive analyses, mean and standard deviation values were used for normally distributed data and median (minimum–maximum) values for non-normally distributed data. Multiple regression models were constructed to determine regression coefficients and 95% confidence intervals for the relationships between body awareness and age, number of pregnancies, gestational weeks, and physical activity. IBM SPSS Statistics v. 29.0 (IBM SPSS Statistics for Windows, Version 29.0. Armonk, NY: IBM Corp.) was used for statistical analyses. The significance level was accepted as p<0.05.

## RESULTS

A total of 149 pregnant women with a mean age of 27.69±4.82 years were included in the study. The mean BMI of the participants was 26.59±3.49; the number of pregnancies was 1.67±0.68; the number of live births was 0.70±0.74, and the gestational weeks were 31.97±6.95. Among the participants, 124 (83.2%) were in the third trimester. The educational status of 115 (77.2%) of the participants was high school. The income status of 116 (77.9%) of the participants was income equal expenditure, while 106 (71.1%) were not employed (Table 1). Of the participants, 76 (51.0%) had a moderate physical activity level. Participants’ body awareness and physical activity levels are given in Table 1.

**Table 1:** Participants’ sociodemographic information, body awareness, and physical activity levels.

		X±SD/n (%)
Age		27.69±4.82
BMI		26.59±3.49
Number of pregnancies		1.67±0.68
Number of live births		0.70±0.74
Gestational weeks		31.97±6.95
Trimester		
	Second	25 (16.8)
	Third	124 (83.2)
Body awareness		92.52±15.48
Physical activity (MET*min/W)		1,084.56±671.93
Physical activity levels		
	Low	64 (43.0)
	Moderate	76 (51.0)
	High	9 (6.0)
Educational status		
	Primary school	6 (4.0)
	Middle school	15 (10.1)
	High School	115 (77.2)
	University and above	13 (8.7)
Income status		
	Income less than expenditure	16 (10.7)
	Income equal expenditure	116 (77.9)
	Income higher than expenditure	17 (11.4)
Employment status		
	Yes	43 (28.9)
	No	106 (71.1)

X: mean; SD: standard deviation; n: number; BMI: body mass index; MET: metabolic equivalent of task.

A multiple linear regression was used to test to predict body awareness based on age, number of pregnancies, gestational weeks, and physical activity. A significant regression equation was found (F=4.363, p=0.002), with an R^2^adj of 0.083. It was found gestational week significantly predicted body awareness (β=0.327, p<0.001, $n_{p}^{2}$ =0.366) (Table 2).

**Table 2:** Multiple regression analysis for age, number of pregnancies, gestational week, and physical activity independent variables.

Variables	Multiple regression analysis for body awareness					
	B (SE)	Lower 95%CI for B	Upper 95%CI for B	Beta	p	Effect size^a^
Intercept	108.800	92.214	125.386		**0.002**	0.108
Age	0.074	-0.537	0.684	0.023	0.812	<0.001
Number of pregnancies	1.612	-2.377	5.601	0.071	0.426	0.004
Gestational weeks	-0.729	-1.089	-0.369	-0.327	**<0.001**	0.100
Physical activity	0.002	-0.002	0.006	0.092	0.295	0.008

B: unstandardized coefficients; Beta: standardized coefficient (SC); R^2^: coefficient of determination; SE: standard error; CI: confidence interval.

a Partial eta squared effect size ( $n_{p}^{2}$ );

p<0.05 is considered as statistically significant (bold).

## DISCUSSION

This study was conducted to examine the relationship between body awareness and physical activity levels, age, number of pregnancies, and gestational weeks in pregnant women. The results of this study showed that in a multiple linear regression analysis to examine the predictive power of age, number of pregnancies, gestational week, and physical activity on body awareness, the model was significant, and gestational week was a significant predictor of body awareness. According to the results of the study, most of the participants were in the third trimester of pregnancy and had a moderate physical activity level.

Pregnancy can cause women to become more sensitive to changes in their bodies and lead to increased body awareness. During pregnancy, there is an increase in women's awareness to recognize the changing needs of their bodies^18^. Physical changes during pregnancy can affect pregnant women's body image, and as the trimesters increase, some women may be affected by these changes and develop negative body image^19^. Experience is important in the processing of body awareness^20^. Therefore, past pregnancy experiences may have an impact on body awareness. The results of this study show that participants had less than two pregnancies on average. This may explain the low impact on body awareness. Body awareness is at its lowest in the third trimester of pregnancy^5^. In the third trimester, which is the last period of pregnancy, the fact that pregnant women are preparing for the role of motherhood may also cause changes in body perception. The findings of the study are similar to the literature. It is thought that the change in body image perception during pregnancy according to trimesters is related to the effect of both psychological and physical changes.

In the literature, it has been reported that physical activity levels of pregnant women are generally inadequate and there is a decrease in physical activity levels of women depending on the trimesters of pregnancy^21,22^. Approximately half (45.9%) of pregnant women were reported to be sedentary^23^. There are a limited number of studies in the literature examining the relationship between physical activity and body image among pregnant women, and the results of the studies vary^24,25^. It is thought that physical problems such as weight gain, fatigue, sleep disorders, and emotional changes during pregnancy may cause changes in the physical activity level; differences in study results may be due to the fact that pregnancy is an individual experience. The results of this study showed that most of the participants had a moderate physical activity level. It is suggested that the fact that most of the participants had moderate physical activity level may lead to inadequate prediction of body awareness.

The body of women who are pregnant for the first time is affected in many ways. Conversations between women and health professionals about women's body image in the early stages of pregnancy can contribute to increased body knowledge, which can have a positive impact in the later stages of pregnancy and in relation to childbirth^13^. There is no study in the literature examining the effect of number of pregnancies on body awareness. The results of this study showed that the number of pregnancies did not predict body awareness. Considering that each birth has a different nature from each other, we think that the number of pregnancies may be the reason why the number of pregnancies does not predict body awareness.

There is no study examining the relationship between age and body awareness in pregnant women. Somatic signaling decreases with aging^26^. Decreasing somatic signals may lead to a decrease in body awareness. The results of this study showed that age did not predict body awareness in pregnancy. We think that the reason for this result is that the pregnant women included in the study were in the age group where there would not be significant decreases in the rate of decline of somatic signals.

## CONCLUSION

This study highlights the significant impact of gestational weeks on body awareness in pregnant women, showing a decline in body awareness as pregnancy progresses. Although no significant associations were found between body awareness and age, number of pregnancies, or physical activity levels, the findings emphasize the importance of addressing body awareness in prenatal care, particularly in the later stages of pregnancy. It is recommended that health professionals integrate interventions to increase body awareness into prenatal care programs, especially in the later stages of pregnancy.

### Limitations

This study had some limitations. One of the limitations of this study is the inclusion of pregnant women in the 2nd and 3rd trimesters. Since it is well known that physical activity levels tend to decrease in the third trimester, future studies may consider including only women in the second or third trimester to obtain more homogenous results. Another limitation was the use of the IPAQ-SF instead of the pregnancy-specific "pregnancy physical activity questionnaire" to determine physical activity levels. This study focused on gestational week, age, number of pregnancies, and physical activity, while other factors that may affect body awareness such as psychological health, social support, and antenatal education were not included. Future research could address these limitations by utilizing a longitudinal design involving a more diverse and representative sample.

## Data Availability

The datasets generated and/or analyzed during the current study are available from the corresponding author upon reasonable request.
